# Phosphine Toxicity: McDaniel et al. Respond

**Published:** 2006-02

**Authors:** Patricia A. McDaniel, Gina Solomon, Ruth E. Malone

**Affiliations:** Center for Tobacco Control Research and Education, University of California, San Francisco, California, E-mail: patricia.mcdaniel@ucsf.edu; Natural Resources Defense Council, San Francisco, California; Department of Social and Behavioral Sciences & School of Nursing, University of California, San Francisco, California

We appreciate the opportunity to respond to Anderson’s letter. We do thank Anderson for providing details on the review process for the article eventually published in *Risk Analysis* (Pepelko et al. 2004) that was based on Sciences International’s work for the Phosphine Coalition. In our article (McDaniel et al. 2005), we did not accuse Anderson of improperly using her status as editor-in-chief of the journal in the publication process. Instead, we point out that she suggested—in a 1999 memorandum to Phosphine Coalition member Joel Seckar—that the peer-review process for the paper could be expedited (Anderson 1999). We then pointed out that the article was ultimately published in 2004. We did not conclude from this that the paper was improperly handled. Indeed, we assumed that it was not, given the 5-year delay between the 1999 proposal and the 2004 publication date. Anderson notes that “it is not uncommon for journals to expedite articles that are of timely interest.” However, we would question whether it is or should be accepted practice for editors who are also authors to initiate an expedited process for their own papers, or to suggest that they would be willing to do so in order to advance the interests of a regulated industry that has hired them in the context of regulatory deliberations.

In addition, we did not state in our article (McDaniel et al. 2005) that the work done by Sciences International led the U.S. Environmental Protection Agency (EPA) to make an improper decision about phosphine risk. We stated that the Phosphine Coalition hired Sciences International to write a report challenging the scientific basis of the U.S. EPA’s proposed risk mitigation measures, focusing on reducing or eliminating the interspecies uncertainty factor that led to the U.S. EPA’s proposed exposure level of 0.03 ppm (Seckar 1999). Sciences International did so; we offered evidence to show that *a*) an early draft was deemed too uncertain and tentative by members of the Phosphine Coalition and was revised by Sciences International to strengthen the language; *b*) the interim report submitted by Sciences International to the U.S. EPA was judged in a memorandum by a U.S. EPA toxicologist (Barolo 1999; Sciences International 1999a, 1999b) to lack the human data necessary to justify eliminating the interspecies uncertainty factor (Whalan 1999); *c*) the U.S. EPA made its final decision on the risk mitigation measures in December 1999, before receiving the final revised report from Sciences International (Sharp 1999). The conclusion we drew from this evidence, which we believe is reasonable regardless of the outcome of the decision itself, is that the U.S. EPA’s regulatory decision making needs to be more transparent. If the U.S. EPA had provided us with the additional internal documents we requested, we might have been able to better understand how the agency made its final regulatory decision, one that left the existing worker exposure standard in place and failed to add community buffer zones and notification requirements as originally proposed.

Anderson suggests that, in our article (McDaniel et al. 2005), we should have examined such challenging scientific issues as categorical regression and the regional gas-dose model for extrapolating from rat inhalation studies to humans. This was not the focus of our work. We examined cases in which the tobacco industry intervened to influence aspects of the pesticide regulatory process. In the case of phosphine, our focus was on the proposed risk mitigation measures that were deemed of primary concern for the tobacco industry: the more stringent worker exposure standard, the buffer zone, and the notification requirements. All of these public health protections were adamantly opposed by the industry coalition, and none of them survived in the final regulatory decision.

We regret that Anderson attacks *EHP* in responding to our article (McDaniel et al. 2005). Our work underwent several levels of peer review before its publication; obviously, we believe that it advances knowledge regarding important regulatory processes—processes that, for good or ill, are both sociopolitical and scientific as they unfold, and in which we believe many *EHP* readers have interest. As we pointed out in our conclusion, although others have charged that agencies responsible for protecting human health and the environment are unduly influenced by the industries they regulate, it is rare to be able to study this process from the perspective of the regulated industry. The tobacco industry documents provide a unique opportunity to identify tactics that industry applies to a regulatory agency when trying to influence the outcome of a decision. The fact that these documents were prepared at a time when their eventual public disclosure was not anticipated raises their archival evidentiary value above what might be learned from contemporaneous interviews years later with persons whose economic interests were at stake in the events discussed. We stand by our interpretation of the documentary record.
